# Determination of hematobiochemical and fore stomach fluid constituents of llama (*Lama glama*) living in Egypt

**DOI:** 10.1007/s11250-023-03820-z

**Published:** 2023-11-13

**Authors:** Mariam Gamal Zaki, Taher Ahmad Baraka, Mohammed Awny Elkhiat, Mohamed Ragaii Younis, Fatma Abd EL-Fattah Tayeb

**Affiliations:** 1 Department of Internal Medicine and Infectious Diseases, Faculty of Veterinary Medicine, Cairo University, Giza, 12211 Egypt; 2 Central Department for Egyptian Zoos and Wildlife Conservation, Giza Zoo, Giza, Egypt

**Keywords:** Llama (*Lama glama*), Hematology, Serum biochemistry, C1 fluid, Sex, Season

## Abstract

There are no available data regarding the hematology, serum biochemistry, and fore stomach fluid constituents of llama (*Lama glama*) in Egypt. This study aimed to establish normal reference values for blood and fore stomach fluid constituents of llama and determine the influence of sex and season on these parameters under Egyptian conditions. The study was performed on (*n* = 38; 22 female, 16 male; 1–7 years) apparently healthy llamas located in the Giza Zoo and private zoo in the Ismailia Governorate. Samples were collected in two seasons and divided into summer and winter samples. Differences in the mean and range values of packed cell volume, serum minerals, fore stomach fluid pH, and total protozoal count in Egypt were recorded. Sex and season had minimal effects on hematology and only erythrocyte count showed a significant (*p* < 0.05) increase in males compared with females. Regarding serum biochemistry, males showed significant (*p* < 0.05) increases in alanine transaminase and calcium levels, while globulin significantly (*p* < 0.05) increased in females. The influence of season on serum biochemistry was evident in alanine transaminase, total protein, albumin, and chloride which increased significantly (*p* < 0.05) in summer, while urea, bilirubin, and magnesium increased significantly (*p* < 0.05) in winter. Fore stomach fluid pH and ammonia showed significant (*p* < 0.05) increases in winter, while the total protozoal count increased significantly (*p* < 0.05) in summer and in males compared with females. The results obtained in this study can serve as reference values for the hematobiochemical and fore stomach fluid constituents of llama in Egypt.

## Introduction

Llama (*Lama glama*) belongs to the family Camelidae, genus *Lama*, and species *Lama glama*. It is one of South American camelids (SAC) that consists of four species: guanaco (*Lama guanicoe*), vicuña (*Vicugna vicugna*), llama (*Lama glama*), and alpaca (*Vicugna pacos*). It is the domestic species of guanaco, which is considered the wild ancestor of this animal species (Kadwell et al., 2001). Llamas are herbivorous animals but not true ruminants as they have three compartment stomach C1, C2, and C3 (C1 is similar to the rumen in true ruminants) (San Martin and Bryant, 1989; Millar et al., 2017). They have a better capacity for digestion of low-quality forages and more efficient extraction of energy and protein than other ruminants (San Martin and Bryant, 1989; Abdou et al., 2006; Ortiz-Chura et al., 2018). Llama is a very important part of the economy in South American countries such as Bolivia, Chile, Argentina, Peru, and Ecuador, and it is widely used as a pack and meat animal and in production of high-quality fiber (Burton et al., 2003). It has adapted to live at high altitudes of 4000 m or more above sea level; however, in recent decades, it was introduced to new environments, at sea level in many countries in South America, Europe, North America, Africa, and Asia (Azwai et al., 2007). Llamas are sociable animals and like living with each other in herds. The number and popularity of llamas have been increasingly growing in Egypt, and they are usually used as show or pet animals in different zoos and farms in the country.

One of the most important methods of monitoring health status is the evaluation of blood and fore stomach fluid constituents of animals with consideration of the influence of intrinsic and extrinsic factors such as sex, nutrition, season, climate, and environment to interpret the results correctly (Abd Al-Galeel et al., 2023). The results will help in the interpretation, diagnosis, and management of clinical and subclinical nutritional disturbances and metabolic health problems (Zapata et al., 2003; Husakova et al., 2014, 2015). There are studies of hematobiochemical and first compartment (C1) fluid parameters of llamas under European, South, and North American conditions (Davies et al., 2007; Del Valle et al., 2008; Foster et al., 2009; Fowler, 2010; Teare, 2013; Ceron Cucchi et al., 2016; Ortiz-Chura et al., 2018); however, to our knowledge, there is no available literature of normal values of blood and C1 fluid parameters of llama in Egypt. The values described for llamas bred in South America and Europe cannot necessarily be applicable to animals bred in Egypt due to geographical, climatic, and nutritional changes. As previous studies on llama focused on blood or C1 fluid constituents separately, our study tried to provide a thorough estimate of the hematobiochemical and C1 fluid profile. Therefore, the objective of our study was to establish the first reference values for hematology, serum biochemistry, and C1 fluid constituents of clinically healthy llamas bred in Egypt with special consideration of the effect of sex and season.

## Material and methods

### Animals

The study was conducted on 38 apparently healthy animals divided into 22 females and 16 males, whose age and body weight ranged between 1–7 years (1–6 years in male group and 1–7 years in female group) and 80–150 kg, respectively. The animals were kept in the Giza Zoo (17 animals) and private zoo in the Ismailia Governorate (21 animals). Samples were collected from July 2022 to February 2023 and divided into summer (from July 2022 to October 2022) and winter (from November 2022 to February 2023) samples. The two zoos had similar management systems of the animals. The animals were lived in large yards, and there were shading (wooden barn) areas to protect the animals from hot sunny and rainy days. Clean water was available all day ad libitum. The two zoos had also the same feeding pattern. The diet was high-quality forages like green alfalfa in winter and hay or dried alfalfa in summer with small intake of grasses or leafs like lettuce and small pieces of carrots as treats. Four animals were included in the study in summer, and they were excluded in winter because they became pregnant or diseased; therefore, they were replaced by other four healthy members had the same age and sex in the herd. All animals in the study were kept under the same environmental, nutritional, and management conditions. The environmental temperature (T) in Egypt during the course of the study ranged from 33 to 40 °C with an average of 36.5 °C in summer, while in winter, it ranged from 17 to 25 °C with an average of 21 °C. An individual comprehensive physical examination was performed on each animal prior to sampling. All animals included in our study were on regular deworming programs, and fecal samples were collected from each animal to test for internal parasites by using direct smear and concentration flotation and sedimentation techniques.

### Sample collection

Samples were taken from animals in early morning without sedation, and only minimum restraint was used. Blood samples were collected via jugular vein puncture using a 10-cm needle, and they were divided into two portions: one portion in EDTA tubes for hematological analysis and the second portion in plain tubes for serum biochemical estimation. C1 fluid (40 ml) was collected by a rubber stomach tube as described by Ceron Cucchi et al. (2016) in sterile cups. Samples were placed in an ice box for transportation to the laboratory.

### Hematological analysis

Hematological examination was performed by the methods of Schalm et al. (1975) and Peinado et al. (1999). The hemoglobin concentration was measured using colorimetric estimation by Drabkin’s solution supplied by Spectrum Company, Egypt. Packed cell volume (PCV) was determined by centrifugation of blood using heparinized capillary tubes and a microhematocrit centrifuge. The total leukocyte and erythrocyte counts were determined using Neubauer’s chamber of the hemocytometer. Differential leukocyte counts were determined by fixing fresh blood smears with methyl alcohol and staining them with Diff-Quick III Stain (Wagener et al., 2021; Abd Al-Galeel et al., 2023). Erythrocyte indices were calculated according to formulas described by Wintrobe (2008).

### Serum biochemical estimation

Serum biochemical analysis was performed by centrifugation of blood samples taken on plain tubes at 3000 rpm for 10 min, and then serum was separated and stored at −20 °C until analysis. The analysis included aspartate aminotransferase (AST), alanine transaminase (ALT), alkaline phosphatase (ALP), lactate dehydrogenase (LDH), gamma-glutamyl transferase (GGT), creatinine, urea, bilirubin, cholesterol, triglyceride, glucose, total protein, albumin, phosphorus, magnesium, calcium, chloride, sodium, and potassium. Globulin and the albumin/globulin ratio were calculated mathematically. All biochemical analyses were performed by Robonik biochemistry analyzer, India, by using specific kits produced by Spectrum Company, Egypt.

### C1 fluid analysis

C1 fluid samples were taken immediately to the laboratory and examined physically (color, odor, consistency), biochemically (pH, ammonia, total and fractionation of volatile fatty acids, calcium, phosphorus, and magnesium), and microscopically (protozoal activity and total protozoal count (TPC)) as described by Al-Azazi et al. (2018) and Zaki et al. (2021a, 2021b). Volatile fatty acid fractionation was performed by HPLC YL 9100, Korea. Estimation of methane and Co_2_ production and fermentation efficiency was determined using formulas described by de OLIVEIRA et al. (2022) and Wang et al. (2022), respectively. Laboratory work was performed at the Department of Internal Medicine, Faculty of Veterinary Medicine, Cairo University.

### Statistics

Statistical analysis was performed by SPSS program version 25. Descriptive statistics (mean, range, and standard error) and normality were determined by using the Shapiro–Wilk test. Independent sample *t* test was used for comparison of normally distributed data of season and sex, while the Mann–Whitney test was used for analysis of non-normally distributed data (phosphorus, mean corpuscular hemoglobin, and protozoal activity). Data are expressed as the mean ± SE, and *p* < 0.05 was considered statistically significant.

## Results

An individual physical examination was performed on each animal prior to sampling. Four animals were excluded in winter because they showed signs of illness or became pregnant, and they were replaced by other four healthy animals had the same age and sex in the herd. The temperature of the animals ranged between 36.7 and 37.8 °C, and all fecal samples taken to test for internal parasites were negative.

Data on the general mean, range, and the effect of sex and season on hematology, serum biochemistry, and C1 fluid constituents are presented in Tables 1, 2 and 3, respectively.

**Table 1:** Hematological values of llama under effect of sex and season in Egypt (*n* = 38; 22 female, 16 male)

Parameter	Sex		Season		General mean	Range
	Female	Male	Summer	Winter		
RBCs (×10^6^)	11.98 ± 0.50	13.61 ± 0.59*	12.16 ± 0.54	13.22 ± 0.58	12.66 ± 0.40	7.9–16.7
Hb (g/dl)	12.40 ± 0.53	13 ± 0.74	12.86 ± 0.60	12.46 ± 0.64	12.66 ± 0.43	8.27–17.46
PCV (%)	26.85 ± 0.62	28.12 ± 0.92	27.55 ± 0.77	27.27 ± 0.77	27.41 ± 0.54	20–33
MCV (fL)	23.39 ± 0.94	22.09 ± 0.73	23.35 ± 1.04	22.27 ± 0.65	22.83 ± 0.62	16.56–31.46
MCH (pg)	10.99 ± 0.51	10.44 ± 0.56	10.88 ± 0.66	10.61 ± 0.37	10.75 ± 0.38	7.39–15.84
MCHC (g/dl)	45.87 ± 1.34	45.99 ± 1.80	46.52 ± 1.61	45.30 ± 1.44	45.93 ± 1.07	31.5–57.79
WBCs (×10^3^)	13.21 ± 0.91	13.48 ± 1.11	12.70 ± 1.14	14.03 ± 0.76	13.33 ± 0.7	5.26–20.75
Relative neutrophil (%)	62.40 ± 1.60	64.93 ± 2.30	64.88 ± 1.63	62 ± 2.26	63.48 ± 1.38	42–76
Absolute neutrophil (×10^3^)	8.79 ± 0.76	8.98 ± 0.87	8.36 ± 0.80	9.42 ± 0.79	8.87 ± 0.56	3.25–15.37
Relative lymphocytes (%)	32.15 ± 1.41	32 ± 2.11	32.11 ± 1.39	33.17 ± 2	32.64 ± 1.20	22–50
Absolute lymphocytes(×10^3^)	4.26 ± 0.35	4.09 ± 0.35	3.86 ± 0.37	4.53 ± 0.32	4.19 ± 0.25	0.84–7.44
Relative eosinophils (%)	2.57 ± 0.51	1.62 ± 37	1.78 ± 0.40	2.55 ± 0.55	2.16 ± 0.34	0–8
Absolute eosinophils (×10^3^)	0.34 ± 0.08	0.21 ± 0.05	0.20 ± 0.05	0.38 ± 0.08	0.29 ± 0.05	0–1.48
Relative basophils (%)	0.38 ± 0.22	0	0.10 ± 0.10	0.33 ± 0.24	0.21 ± 0.12	0–4
Absolute basophils (×10^3^)	0.05 ± 0.03	0	0.01 ± 0.01	0.04 ± 0.03	0.02 ± 0.1	0–0.61
Relative monocytes (%)	2.19 ± 0.49	1.37 ± 0.50	1.78 ± 0.48	1.88 ± 0.54	1.83 ± 0.35	0–8
Absolute monocytes (×10^3^)	0.30 ± 0.06	0.17 ± 0.04	0.24 ± 0.06	0.24 ± 0.05	0.24 ± 0.04	0–0.38

Data are expressed as the mean ± SE. Asterisk (*) in the row means significant difference (*p* < 0.05) in sex and season

**Table 2:** Serum biochemical values of llama under effect of sex and season in Egypt (*n* = 38; 22 female, 16 male)

Parameter	Sex		Season		General mean	Range
	Female	Male	Summer	Winter		
AST (IU/L)	146.43 ± 9.22	158.21 ± 9.94	142.59 ± 11.14	160.89 ± 7	151.48 ± 6.75	81.23–239
ALT (IU/L)	1.87 ± 0.26	3.13 ± 0.51*	2.92 ± 0.39*	1.81 ± 0.34	2.38 ± 0.27	0.07–5.99
ALP (IU/L)	103.49 ± 9.39	129.69 ± 14.77	117.80 ± 11.56	109.06 ± 12.11	113.68 ± 8.27	42.99–225.37
LDH (IU/L)	117.56 ± 12.76	99.62 ± 15.64	106.08 ± 13.36	114.74 ± 14.88	110.29 ± 9.86	21.69–223.26
GGT (IU/L)	43.39 ± 1.61	46.72 ± 2.62	44.89 ± 2.40	44.58 ± 1.59	44.74 ± 1.43	24.11–67.38
Creatinine (mg/dl)	2.23 ± 0.08	2.21 ± 0.11	2.30 ± 0.09	2.14 ± 0.09	2.22 ± 0.06	1.63–3.14
Urea (mg/dl)	48.07 ± 3.65	44.96 ± 3.30	37.23 ± 3.28	56.32 ± 2.14*	46.77 ± 2.51	20.84–72.95
Bilirubin (mg/dl)	0.45 ± 0.05	0.56 ± 0.06	0.38 ± 0.04	0.59 ± 0.05*	0.49 ± 0.04	0.12–0.98
Cholesterol (mg/dl)	31.45 ± 2.18	40.61 ± 5.41	33.38 ± 4.43	37.04 ± 2.72	35.16 ± 2.61	11.19–86.13
Triglyceride (mg/dl)	32.31 ± 1.78	33.14 ± 3.69	33.34 ± 2.60	31.92 ± 2.90	32.65 ± 1.80	14.19–59.35
Glucose (mg/dl)	117.30 ± 3.67	115.53 ± 5.81	113.74 ± 5.03	119.58 ± 3.77	116.58 ± 3.16	82.28–159.79
Total protein (g/dl)	5.58 ± 0.14	5.31 ± 0.12	5.70 ± 0.12*	5.23 ± 0.13	5.47 ± 0.10	4.38–7.42
Albumin (g/dl)	3.43 ± 0.09	3.55 ± 0.11	3.71 ± 0.10*	3.24 ± 0.07	3.48 ± 0.07	2.64–4.73
Globulin (g/dl)	2.14 ± 0.15*	1.75 ± 0.07	1.98 ± 0.14	1.99 ± 0.14	1.99 ± 0.10	1.25–3.75
A/G ratio	1.82 ± 0.16	2.09 ± 0.14	2.07 ± 0.18	1.77 ± 0.13	1.93 ± 0.11	0.915–3.78
Phosphorus (mg/dl)	6.7 ± 0.24	7.44 ± 0.54	7.09 ± 0.31	6.91 ± 0.44	7.00 ± 0.26	3.38–10.72
Magnesium (mg/dl)	1.18 ± 0.06	1.20 ± 0.08	1.05 ± 0.05	1.35 ± 0.05*	1.95 ± 0.04	0.7–1.84
Calcium (mg/dl)	10.41 ± 0.25	11.15 ± 0.20*	10.65 ± 0.29	10.76 ± 0.20	10.70 ± 0.18	8.62–12.46
Chloride (mmol/l)	113 ± 41 ± 1.69	114.15 ± 2.07	116.86 ± 1.48*	110.38 ± 1.89	113.71 ± 1.29	98.76–128.46
Sodium (mmol/l)	165.92 ± 6.05	170.33 ± 4.44	170.78 ± 5.05	166.58 ± 5.09	168.68 ± 3.54	128.45–199.15
Potassium (mmol/l)	7.15 ± 0.27	7.28 ± 0.47	7.13 ± 0.34	7.28 ± 0.34	7.2 ± 0.24	5.36–8.97

Data are expressed as the mean ± SE. Asterisk (*) in the row means significant difference (*p* < 0.05) in sex and season

**Table 3:** C1 fluid constituent values of llama under effect of sex and season in Egypt (*n* = 38; 22 female, 16 male)

Parameter	Sex		Season		General mean	Range
	Female	Male	Summer	Winter		
Color	Dark brown to olive green		Dark brown	Olive green		
Odor	Aromatic odor					
Consistency	Slimy					
Protozoal activity	++To +++					
pH	7.32 ± 0.08	7.2 ± 0.10	7.16 ± 0.07	7.53 ± 0.05*	7.28 ± 0.06	6.5–7.7
TVFAs (mmol/l)	50.76 ± 2.95	49.31 ± 4.84	49.15 ± 3.16	53.12 ± 3.80	50.33 ± 2.47	30–72.5
Acetic (mmol/l)	30.82 ± 4.07	28.19 ± 4	29.95 ± 4.28	29.95 ± 4.04	29.95 ± 2.97	13.5–62.05
Propionic (mmol/l)	16.31 ± 1.79	15.30 ± 2.70	14.63 ± 1.71	18.07 ± 2.55	15.97 ± 1.45	5.25–25.9
Buyteric (mmol/l)	10.01 ± 1.78	8.71 ± 1.37	8.66 ± 1.70	10.95 ± 1.50	9.52 ± 1.20	1.27–18.12
Ammonia (mmol/l)	5.08 ± 0.59	5.26 ± 0.74	4.22 ± 0.50	7.08 ± 0.57*	5.14 ± 0.46	1.74–10.12
Phosphorus (mmol/l)	1.01 ± 0.08	0.88 ± 0.14	1.04 ± 0.09	0.82 ± 0.11	0.97 ± 0.07	0.12–1.77
Calcium (mmol/l)	0.56 ± 0.08	0.64 ± 0.08	0.64 ± 0.08	0.47 ± 0.04	0.59 ± 0.06	0.18–1.38
Magnesium (mmol/l)	0.29 ± 0.03	0.38 ± 0.03	0.35 ± 0.03	0.27 ± 0.01	0.32 ± 0.02	0.1–0.55
TPC (10^4^/ml)	13.33 ± 1.49	19.55 ± 1.73*	17.68 ± 1.52*	11.62 ± 1.63	15.66 ± 1.27	6.5–28
Co_2_ (mmol/l)	33.87 ± 3.73	31 ± 4.41	31.75 ± 3.92	34.53 ± 3.89	32.79 ± 2.79	17.21–52
Methane (mmol/l)	16.16 ± 2.45	14.62 ± 1.94	15.82 ± 2.50	15.19 ± 1.83	15.58 ± 1.66	3.9–29
Fermentation efficiency (%)	78.59 ± 1.31	78.29 ± 0.58	78.01 ± 1.44	79.16 ± 0.42	78.48 ± 0.85	69.37–88.09

Data are expressed as the mean ± SE. Asterisk (*) in the row means significant difference (*p* < 0.05) in sex and season

+++ = overcrowded and high motile protozoa; ++ = decrease in motility and/or crowdness of protozoa

*TVFAs* total volatile fatty acids, *TPC* total protozoal count

Hematological parameters data showed a significant (*p* < 0.05) elevation in red blood cells in males compared to their female counterparts, while other parameters did not differ statistically under the effect of sex or season (Table 1).

Serum biochemical parameters showed a significant (*p* < 0.05) increase in ALT and calcium in males, while in females, globulin showed a significant (*p* < 0.05) elevation compared to males. Under the effect of season, llama in the summer season showed significant (*p* < 0.05) elevations in total protein, albumin, and chloride regardless of sex, while in winter, urea, bilirubin, and mg were significantly (*p* < 0.05) increased (Table 2).

Physical, biochemical, and microscopic analyses of C1 fluid (Table 3) showed significantly (*p* < 0.05) higher total protozoal counts in males than in females. C1 fluid color changed from dark brown in summer to olive green in winter. There was a significant (*p* < 0.05) increase in pH and ammonia in winter, while in summer, TPC was significantly (*p* < 0.05) increased.

## Discussion

Llama (*Lama glama*) is a South American camelid that was introduced to Egypt in recent decades and has witnessed a remarkable increase in number and popularity. The environmental and climatic difference between native countries of llama and Egypt necessitates re-establishment of normal reference values of this animal species under Egyptian conditions with special consideration of the effect of sex and season.

The general means of erythrocyte count, hemoglobin (Hb), mean corpuscular volume (MCV), mean corpuscular hemoglobin (MCH), and mean corpuscular hemoglobin concentration (MCHC) in our study were similar to those previously reported for clinically healthy llamas (Al-Izzi et al., 2004; Al-Bassam et al., 2007; Azwai et al., 2007; Foster et al., 2009; Fowler, 2010; Teare, 2013). Erythrocyte counts of SAC are relatively high compared with other compounded stomach animals and have a unique elliptical, flat, and small shape, which facilitates their movement in capillaries during dehydration (Azwai et al., 2007). The small volume of RBCs resulted in a high concentration for any given PCV (Vap and Bohn, 2015). MCHC values for SAC are more than 40 g/dl, which is higher than those of other ruminants, while those of MCV are lower than those of other species, which provides a large surface area/volume ratio for more efficient gas exchange (Foster et al., 2009). The mean and range values of PCV in this study (20–33%) were lower than those in other studies (22.9 to 46%) (Al-Izzi et al., 2004; Abdou et al., 2006; Al-Bassam et al., 2007; Azwai et al., 2007) in Libya and (Foster et al., 2009; Fowler, 2010; Teare, 2013) in Europe and South America. This slight difference in PCV values could be due to a lack of water at high altitudes or in winter season, as it used to be frozen in the morning in South America and Europe (Zapata et al., 2003), or because of climatic and geographic differences between Libya and Egypt (Index Mundi 2023). SAC can appear healthy with a PCV of < 10%, and they are able to tolerate severe anemia (Foster et al., 2009). Our mean and range values of total and differential leucocytes were similar to those of Al-Izzi et al. (2004), Al-Bassam et al. (2007), Azwai et al. (2007), Foster et al. (2009), Fowler (2010), and Teare (2013). The leucogram in SAC is characterized by increasing total leucocyte count accompanied by high neutrophil and eosinophil counts (Azwai et al., 2007) in comparison with other farmed ruminants as reported by Zaki et al. (2021a, 2021b) in sheep and Ramadan et al. (2019) in cattle in Egypt. The effect of sex was minimal on hematological parameters, and only RBC count showed a significant (*p* < 0.05) increase in males compared with females in agreement with Al-Izzi et al. (2004). The hematological parameters of llama were not affected by seasonal changes. Changing in hematological parameters of llama between seasons may need strong change in climate (temperature and humidity) however, this is not the case in Egypt, as summer is hot and dry while winter, albeit rainy, is warm.

In our study, the general mean and range values of enzymes, glucose, urea, creatinine, total protein, albumin, globulin, cholesterol, triglycerides, and bilirubin were similar to Davies et al. (2007), Foster et al. (2009), Fowler (2010), and Teare (2013), while there were obvious variations in values of serum minerals between our study and the abovementioned studies. This difference may be attributed to changes in nutrition, the environment, and soil; however, all values were in the normal range, and all animals were apparently healthy. There was a significant (*p* < 0.05) increase in ALT in males compared with females, in agreement with Faye and Bengoumi (2018) in dromedary camel. This increase could be due to increase metabolic rate and liver transaminase in males due to high testosterone levels (Lin et al., 1959; Boots et al., 1969). ALT values also showed a significant (*p* < 0.05) increase in the summer period compared with the winter period, in line with Husakova et al. (2014), who found a significant increase in parameters related to liver metabolism, such as ALT, in the summer period. Calcium values increased significantly (*p* < 0.05) in males compared with females, which could be explained by the relationship between sex hormones and serum calcium (Koek et al., 2021). Females had significantly higher (*p* < 0.05) globulin values than males in agreement with Patodkar et al. (2010), Elkhair and Hartmann (2014), and Faye and Bengoumi (2018) who returned this increase in globulin levels to the physiological status of females, as albumin may have lower values during the lactation period. However, our study did not include lactating females; therefore, the increase in globulin values could be attributed to the effect of estrogen in females (Khadjeh, 1998). The summer season showed a significant (*p* < 0.05) increase in total protein and albumen, in agreement with Amin et al. (2007) and Abdoun et al. (2012), who suggested that the increase in total protein in the dry season was due to the stress on animals in this period. Summer also had a significant (*p* < 0.05) increase in chloride level in agreement with Faye and Bengoumi (2018), who reported higher chloridemia in dromedary camel during the dry season, which passed from 112 mmol/l to the peak 146 mmol/l and returned to normal level 24 h after rehydration. In the winter season, there was a significant (*p* < 0.05) increase in urea and magnesium levels, and this increase could be related to increased protein and magnesium levels in green forages in winter due to higher quality and availability of forages in green season (Amin et al., 2007; Aichouni et al., 2013; Faye and Bengoumi, 2018). Bilirubin values also showed a significant (*p* < 0.05) increase in winter, which was in contrast to Husakova et al. (2014), who explained that the higher values of bilirubin in the summer season were due to increased liver metabolism. However, the increase in bilirubin levels in winter in our study was within the normal range, which could be attributed to decreased sunshine duration in winter or due to decreased dry matter intake.

Data on the normal physical characteristics of C1 fluid of llama are scarce, and most reports have focused on biochemical constituents, protozoal, and bacterial counts and species (Dulphy et al., 1997; Del Valle et al., 2008; Ceron Cucchi et al., 2016; Ortiz-Chura et al., 2018). The color of C1 fluid changed according to feeding type; in summer, it was dark brown in color, while in winter, it was olive green in color. The odor, consistency, and protozoal activity were similar to those reported by Baraka et al. (2000) and Eissa et al. (2022) in dromedary camel and Al-Azazi et al. (2018) and Zaki et al. (2021a, 2021b) in sheep. The C1 fluid pH of llama in our study was higher than those previously described (Dulphy et al., 1997; Ortiz-Chura et al., 2018); however, it was still within the normal range (6.5–7.5) reported by Nilsen et al. (2015). Dulphy et al. (1997) used four types of ration consisted of different concentrate types, while Ortiz-Chura et al. (2018) mentioned in their study that animals were feed only on low-quality forages; therefore, this difference in pH could be resulted from nutritional divergence or absence of concentrate intake in our study compared with the abovementioned studies. The significant (*p* < 0.05) increase in C1 pH in winter could be explained by increased ammonia level in winter. Ammonia increased in winter due to increased protein intake and increased urea concentration in blood. In Camelidae and ruminants, urea is partly returned to C1 and broken down by microflora to ammonia (Faye and Bengoumi, 2018); therefore, ammonia is expected to increase in winter. The total protozoal count was lower than that reported by Del Valle et al. (2008) in Bolivia; however, it was higher than that reported by Ceron Cucchi et al. (2016) and Ortiz-Chura et al. (2018) in Argentina. This difference in protozoal count may be attributed to changes in feeding type, environment, and C1 ecosystem. TPC count increased significantly (*p* < 0.05) in males compared with females, and this significant increase could be attributed to increased feed consumption in males. TPC count also showed a significant (*p* < 0.05) increase in summer compared with winter as a result of an increase in C1 pH in winter. Ciliates are sensitive to changes in pH, in agreement with Zaki et al. (2021b), who reported a significant decrease in TPC at high rumen pH. The concentrations of total and fractionated volatile fatty acids were similar to those in Ortiz-Chura et al. (2018), and they were not affected by sex or seasonal changes. Co_2_, methane, and fermentation efficiency values were in agreement with Baraka and Abdl-Rahman (2012) and did not differ statistically under the effect of season or sex. There are no data available about macro mineral concentrations in llama C1 fluid. Our values of mineral concentrations in llama C1 fluid were less than those reported by Baraka et al. (2000) and Shoeib et al. (2019) in dromedary camels as a result of differences in animal species, C1 pH, and feeding type.

## Conclusion

Hematology, serum biochemistry, and C1 fluid constituent values of clinically healthy llamas under Egyptian circumstances were different in PCV, serum minerals, C1 fluid pH, and total protozoal count from those of authors in other countries, while the rest of parameters were similar to other reports. The previous results support the theory that reference values of blood and C1 fluid constituents should be re-established regularly to account for gender, climatic, nutritional, geographic, and genetic variations. There was a significant effect of sex and season on serum biochemistry and C1 fluid constituents. The obtained data can be used by veterinarians in Egypt as reference values for blood and C1 fluid constituents of llama to evaluate the health status, diagnosis, and management of clinical and subclinical diseases of this animal species.

## Data Availability

The datasets analyzed during the current study are available from the corresponding author on reasonable request.
